# Cutaneous malignant melanoma incidence is strongly associated with European depigmented skin type regardless of ambient ultraviolet radiation levels: evidence from Worldwide population-based data

**DOI:** 10.3934/publichealth.2022026

**Published:** 2022-03-17

**Authors:** Wenpeng You, Renata Henneberg, Brendon J Coventry, Maciej Henneberg

**Affiliations:** 1 Biological Anthropology and Comparative Anatomy Unit, Adelaide Medical School, the University of Adelaide, Adelaide, South Australia, Australia; 2 Discipline of Surgery, University of Adelaide, Royal Adelaide Hospital, Adelaide, South Australia, Australia; 3 Institute of Evolutionary Medicine, University of Zurich, Zurich, Switzerland

**Keywords:** cutaneous malignant melanoma (CMM), incidence, UV levels, depigmentation, adaptation, world-wide data

## Abstract

Current public health advice is that high ultraviolet radiation (UVR) exposure is the primary cause of Malignant Melanoma of skin (CMM), however, despite the use of sun-blocking products incidence of melanoma is increasing. To investigate the UVR influence on CMM incidence worldwide WHO, United Nations, World Bank databases and literature provided 182 country-speciﬁc melanoma incidence estimates, daily UVR levels, skin colour (EEL), socioeconomic status (GDP PPP), magnitude of reduced natural selection (Ibs), ageing, urbanization, percentage of European descendants (Eu%), and depigmentation (blonde hair colour), for parametric and non-parametric correlations, multivariate regressions and analyses of variance. Worldwide, UVR levels showed negative correlation with melanoma incidence (“rho” = −0.515, p < 0.001), remaining significant and negative in parametric partial correlation (r = −0.513, p < 0.001) with other variables kept constant. After standardising melanoma incidence for Eu%, melanoma correlation with UVR disappeared completely (“rho” = 0.004, p = 0.967, n = 127). The results question classical views that UVR causes melanoma. No correlation between UVR level and melanoma incidence was present when Eu% (depigmented or light skin type) was kept statistically constant, even after adjusting for other known variables. Countries with lower UVR levels and more Eu% (depigmented or light skin people) have higher melanoma incidence. Critically, this means that individual genetic low skin pigmentation factors predict melanoma risk regardless of UVR exposure levels, and even at low-UVR levels.

## 1. Introduction

Malignant melanoma is a cancer particularly common among Europeans [1]. It is a malignancy of melanocytes which are primarily located in the hairy skin (Cutaneous Malignant Melanoma — CMM herefter) [2], but also arise at internal mucosal sites and in glabrous skin (acral). CMM represents a growing public health burden worldwide. Globally, the melanoma incidence rate has been increasing over past decades to reach 4.2 per 100,000 worldwide, with the highest in WHO European region (16.6 per 100,000) in 2020 [3],[4]. Much effort has been expended to investigate the extent to which changes in behaviours, related to exposure to the ultraviolet radiation (UVR), are involved, but the mechanism of the increasing incidence is still not well understood [5].

CMM occurs significantly more often in people of European descent [6]–[10]. The annual increase in CMM incidence rate for European-derived populations remains much higher than for all other populations [6],[11]. In 2020, the incidence rate in the World Health Organization (WHO) European region was 4 times higher than that worldwide [3]. Therefore, typically European phenotypes, such as blond hair, light-coloured eyes, fair skin, more naevi [12] and freckles [13]–[16] have been considered as risk factors for CMM independent of the UVR.

Extensive studies have linked CMM risks to sunlight exposure-related behaviours, such as strong sun exposure in childhood [17],[18], sunburn episodes [19],[20], solarium use [21]–[23], and outdoor work [24]. Although the conclusions are largely associative and circumstantial, high UVR exposure has been commonly accepted as the primary risk factor for CMM. Reported effect sizes of relationships with the UVR exposure, though statistically significant, are small.

It has been postulated that exposure to intense UVR levels causes damage to the DNA of melanocytes, which constitutes the major contributor for developing CMM [25]–[31]. Although this theory has some supportive evidence, it poorly explains the true epidemiology of CMM worldwide and the regional variations. For example, UVR in the Europe region of WHO is significantly lower than in other regions [32], but in Europe melanoma incidence rate is significantly higher than in other WHO regions [33]. In a study of over 50 populations worldwide [34],[35] no relationship between personal UVR exposure and CMM incidence was found.

Directed by the different levels of association between cancer development and virus infections, in particular, the role of human papilloma virus (HPV) in non-CMM skin cancer initiation [36], HPV, as a sexually transmitted disease, has been associated with CMM development because it could create insertional mutagenesis in human DNA of melanocytes [37]–[40]. The strongest evidence for that is the association between exponential increase of CMM incidence and the sex revolution in Europe after 1960 [38],[39].

CMM prevention campaigns have advocated application of sunscreen to reduce the CMM risk [41]. However, some epidemiological studies have shown that sun-blocking substances, such as sunscreen lotion, do not protect against CMM development [42]–[44]. Surprisingly, and paradoxically, individuals regularly applying sun block may have a higher risk of CMM than non-users [43],[45]–[47]. Therefore, the role of sun-blocking products and their application in protecting against melanoma development remains confusing and intriguing [5],[43],[48].

There is a debate over whether intermittent [49] or chronic [50] sun exposure represent important risks for melanoma. Part of this debate may seem pointless because CMM subtypes can develop in deep skin areas with little or no UVR exposure in humans (i.e. mouth, soles of feet, palms of hands, mucosal sites, buttocks and genital areas) [1],[51]. Furthermore, in hairy skin areas, intermittent, rather than chronic, UVR exposure has been postulated as a major risk for CMM as it reduces the penetration of UVR into the skin [52]. Low UVR exposure also causes CMM in animals, for example in the dark fur covered areas of canines [53] and in mice with various depigmentation phenotypes [54]. In recent systematic survey of literature no relationship has been found between moderate solarium exposure and melanoma risk [55]. Moreover, occurrence of some melanoma subtypes (e.g. acral lentiginous) is clearly not related to UVR exposure. The whole-genome mutational profiles of some melanoma subtypes that occur without sun exposure possibly extend to the non-coding genome [1],[56]. It has been also reported that UVR would not be the only determinant for CMM if people carried MC1R genes [56].

Regardless of rare early CMM onset in young people with fair pigmentation [57], ageing is a well-established risk factor, which may be partly attributable to the accumulated detrimental effects of sun exposure [13],[17],[58].

From an evolutionary perspective, it has been known that human adaptation to low UVR exposure in Northern Europe over many generations resulted in decreased melanin production, most likely to permit sufficient vitamin D production in the skin in these environments [59]. During this evolutionary process, recessive mutations/genes interfering with normal melanin production for skin, hair and eye colouration were accumulated. This process may also be advantageous to the levels of folate and folic acid in human blood [60]–[62].

Interestingly, CMM incidence in predominantly European (reduced skin pigment) populations in low ambient UV regions is higher than would be expected, if high ambient UV levels alone were the principal determinant of CMM development. Reduced skin pigmentation in itself might therefore be a factor for CMM development.

Therefore in this study, the discrepancies in the UVR and CMM relationship were investigated for their association and relationship trend. We advance the hypothesis that evolutionary processes leading to strongly reduced ability to produce melanin, may, as a side effect, foster somatic mutations resulting in CMM development. This hypothesis is tested using worldwide population-level data on human pigmentation and CMM incidence obtained from international data-collection organizations. These data were adjusted for ambient UVR levels and other potential confounding variables.

## 2. Materials and methods

### 2.1. Data sources

Country-specific data published by the agencies of the United Nations were downloaded for this ecological study.

1. The GLOBOCAN 2012 estimate of country specific melanoma of the skin (WHO ICD: C43, CMM as abbreviated previously) incidence rate in both sexes [3],[33].

GLOBOCAN provides contemporary population level estimates by cancer site and sex [63]. This project is conducted by the World Health Organization cancer research agency, the International Agency for Research on Cancer (IARC).

As per the International Statistical Classification of Diseases and Related Health Problems (10th Revision (ICD-10)-2015-WHO Version for 2015), IARC clustered 10-types of malignant neoplasms of skin as malignant melanoma of skin which is coded as C43.

CMM incidence rate is expressed as the number of persons who were diagnosed with CMM per 100,000 population. The CMM incidence reported as an age standardised rate at world level was selected for analyses. No incidence of separate types or subtypes of CMM was available.

2. Country-specific skin colour measured by reflectance (armpit). Data on skin reflectance of various populations worldwide were previously studied and published [60],[61],[64],[65]. We extracted the country-specific skin reflectance data relevant for each country from previous publications using the same file as analysed in Brace et al. [66]. Worldwide information on skin phototypes was not available.

3. The WHO Global Health Observatory (GHO) data on the average daily ambient ultraviolet radiation (UVR) level (in J/m^2^) [32] and life expectancy at age 60-years [67]. UVR has been backdated by approximately 10 years (1997−2003) to reflect long exposure duration with delayed presentation of CMM.

Ageing has been included as a potential confounder in this study as it has been linked to CMM risk in a number of publications [13],[17],[58]. We have indexed ageing at the population level with the life expectancy at age 60 in 2010.

4. The World Bank published data on per-capita GDP PPP and urbanization [68].

Socio-economic level has been associated with CMM risk [63],[69]. We have chosen per capita GDP purchasing power rate (GDP PPP in 2012 international $) because it takes into account the relative cost of local goods, services and inflation rates of the country.

Urbanization has been postulated as a major CMM predictor [70] because it represents the major demographic shift entailing lifestyle changes [71]–[73]. Urbanization is expressed with the country-specific percentage of total population living in urban areas in 2012.

5. Country-specific magnitude of possible CMM gene accumulation downloaded from a previous publication [74]. This accumulation is assumed to be the effect of decreasing selection pressure that is changing mutation/selection balance. The Biological State Index (I_bs_) has been constructed to measure the opportunity for natural selection at the population level [61–67 and the Supplementary Information].

6. The CMM incidence rates vary between geographical areas, with the highest rates in Europe [33],[63] and in countries with the greatest proportion of European descendants [6],[11],[75]–[77]. Therefore, we have constructed the following two further variables:

1) Country-specific percentage of European descendants (Eu% hereinafter) was collected from the EuroStat for European countries [78], and government and non-EU government documents for the rest of the countries with European descendants.

2) The country grouping of the WHO Europe Region was singled out for analysing the correlation between UVR and CMM. We also obtained the country-specific percentage of population with light hair [79] as the measurement of the magnitude of depigmentation (depigmentation level hereafter).

All the known potential confounding variables (GDP PPP, I_bs_, ageing and urbanization) and independent variables (skin reflectance, UVR, Europeans % and depigmentation level) were matched with the dependent variable, country specific CMM incidence rate to reduce potential bias. A set of data consisting of 182 countries has been obtained for our analysis. Each country was treated as an individual subject in this study. The number of countries for each individual variable may differ because not all the countries had uniformly available information due to various reasons.

### 2.2. Data analysis

**Figure 1.**
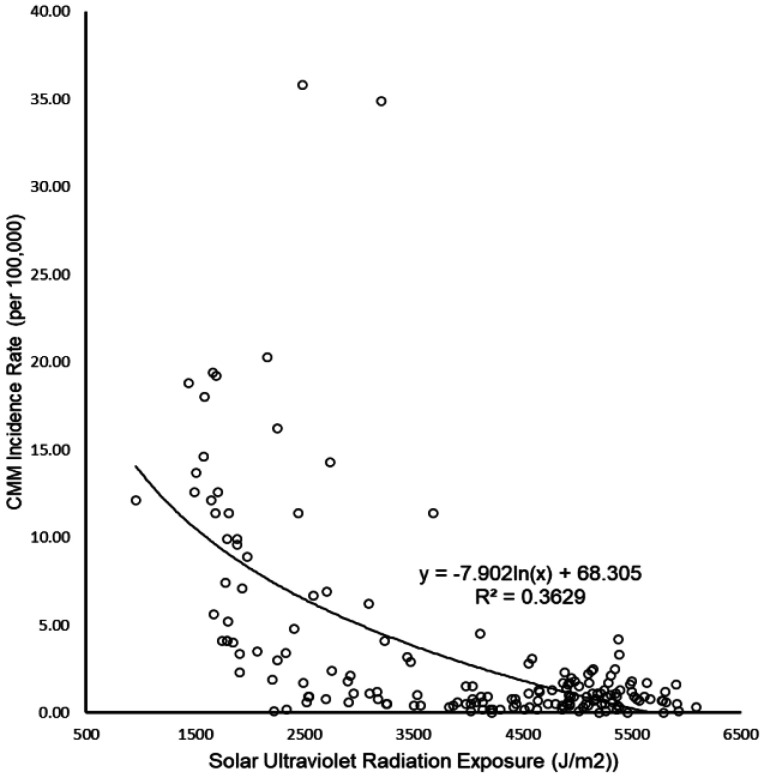
Relationship (inverse correlation) between solar ultraviolet radiation exposure and cutaneous malignant melanoma (CMM) of skin cancer incidence rate worldwide.

With reference to the conceptual framework of the data analysis in the previous studies [80]–[90], the data analysis proceeded in six steps:

1. Scatter plots were produced with the cross-country raw data in Microsoft Excel^®^ to explore and visualize the strength, shape and direction of correlation between UVR levels and CMM incidence worldwide. Points representing Australia and New Zealand appeared to be the outliers (Figure 1). However, we did not remove them because they represented the truth that Australia and New Zealand have had the highest CMM incidence rates although their UVR levels have not been the highest [6],[11],[91]. Scatter plots were also produced to explore the relationships between the CMM incidence and Europeans % worldwide, country-specific UVR within WHO-Europe and depigmentation level within the European area respectively.

2. Nonparametric correlation analysis (Spearman's ρ) was conducted to evaluate the worldwide direction and strength of the correlation between CMM and each independent and potentially confounding variable.

3. Partial correlation of Pearson's moment-product approach on log-transformed data was conducted to explore the worldwide correlations between CMM and UVR and CMM and skin reflectance respectively when we controlled for the potential confounding variables (GDP PPP, I_bs_, ageing and urbanization).

4. Standard multiple linear regression (Stepwise) was conducted on log-transformed variables to select the variables that had the greatest influence on CMM incidence when UVR, I_bs_, ageing, GDP PPP and Urbanization were entered as the independent variables.

Considering that CMM has been associated with people of European origin, we replaced UVR with the other two variables, “WHO EU Region” and “Europeans %”, respectively and repeated the above analyses (Step 2–4). In these two subsequent analyses, we did not analyse the relationship between the variable of skin reflectance and CMM due to the very limited number of countries with available data in each data set.

Additionally, when we conducted the partial correlation within the dataset of “WHO EU Region”, we alternated depigmentation and UVR as the control variable together with the other confounding variables (GDP PPP, I_bs_, ageing and urbanization) to explore whether the variables UVR and depigmentation were correlated with CMM incidence independent of each other. Similarly, when we conducted the partial correlation with the dataset of “Europeans %”, we alternated Europeans % and UVR as the control variables together with the other confounding variables (GDP PPP, I_bs_, ageing and urbanization) to explore whether the variables UVR and EU% correlated with CMM independent of each other.

5. Analysis of variance (ANOVA) was conducted to detect the significant differences between the six WHO regions among the means of CMM, and “Residual of CMM standardised on UVR” [92]. Further post-hoc (Bonferroni) testing was performed to identify the source (pairs) of the significant differences.

6. European population (WHO European Region) has the significantly higher incidence of CMM [6],[11],[75],[76], but significantly lower UVR levels than in all the other WHO regions. To examine whether, statistically, they can explain each other in terms of their worldwide relationships, we have used the analysis of residuals, because CMM incidence is curvilinearly related to UVR and Europeans %. Details in Supplementary Information.

All analyses were conducted on SPSS v. 25. The significance was reported at 0.05, 0.01 and 0.001 levels. Standard multiple linear regression analysis criteria were set at probability of F to enter ≤ 0.05 and probability of F to remove ≥ 0.10.

## 3. Results

### 3.1. UVR and CMM

Worldwide, the relationship between UVR and CMM, identified in the scatterplots was noted to be logarithmic with a relatively strong, but *negative* correlation (r = −0.60, p < 0.001, n = 171, Figure 1). This indicates that people living in low solar ultraviolet radiation environments have higher CMM incidence.

Worldwide, UVR intensity was in significant and negative correlation with CMM in non-parametric correlation analysis (r = −0.52, p < 0.001, Table 1–1). This relationship remained negative and significant in partial correlation (r = −0.51, p < 0.001) when GDP PPP, I_bs_, ageing and urbanization were statistically kept constant (Table 1–2).

Skin reflectance correlated positively with CMM [lighter skin-more CMM] at a marginally significant level (r = 0.33, p = 0.057, n = 35, Table 1–1). This correlation became weak (r = 0.15, p = 0.505, df =19) when GDP PPP, I_bs_, ageing and urbanization were statistically kept constant (Table 1–2).

When UVR, GDP PPP, I_bs_, ageing and urbanization were included as the predictor variables in stepwise linear regression analysis, UVR was selected as the variable having the greatest negative influence on CMM incidence rate (R^2^ = 0.30, Table 1–3).

**Table 1.** Worldwide relationships between melanoma (CMM) incidence and UVR levels.

	Table 1–1: Nonparametric (Spearman's)		Table 1–2: Partial Correlation^#^		Table 1–3: Stepwise multiple linear regression		
	ρ	n	r	df	Rank	Independent Variables	Adjusted R^2^
UVR exposure (Negative)	−0.515***	171	−0.513***	163	1	UVR Exposure (Negative)	0.301
Skin reflectance	0.325^##^	35	0.153	19	2	I_bs_	0.321
GDP PPP	0.383***	172	-	-	3	Ageing	0.363
I_bs_	0.456***	172	-	-	4	GDP PPP	Insignificant
Ageing	0.415***	174	-	-	5	Urbanization	Insignificant
Urbanization	0.354***	178	-	-			

*Note: Significance level of correlation: *p < 0.05, **p <0 .01, ***p < 0.001. ^#^Keeping intake of GDP PPP, I_bs_, life e_(60)_ and urbanization constant.^##^p = 0.057, marginally significant at the level of p < 0.05. Data sources: Melanoma of skin incidence rate from the International Agency for Research on Cancer, WHO agent in cancer research; UVR, expressed as the average daily ambient ultraviolet radiation level (in J/m^2^) & ageing, indexed by life e60 from the World Health Organization; Skin reflectance from previous publication (See the section of Data Sources please); GDP PPP & Urbanization from the World Bank; Ibs from the previous publication (See the section of Data Sources please).

**Figure 2.**
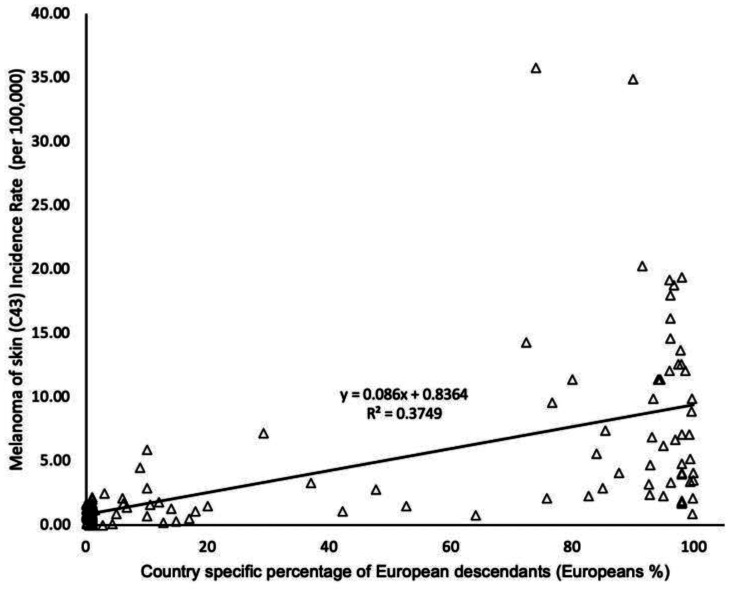
Worldwide relationship between country specific percentage of European descendants and cutaneous malignant melanoma (CMM) of skin cancer incidence rate.

The ANOVA has revealed that WHO EU Region had significantly the highest mean CMM incidence rate among other WHO regions while having the significantly lowest mean of UVR (2189 J/m^2^). There were no significant differences between other regions (Table S1).

Worldwide, the relationship between Europeans % and CMM in the scatterplots was linear, with a positive correlation (r = 0.61, p < 0.001, n = 135, Figure 2).

### 3.2. Effect of % Europeans/ depigmentation

Table 2–1 shows that CMM incidence is in positive strong correlation with Europeans % (r = 0.71, p < 0.001) and in similarly strong, but in negative correlation with UVR levels (r = −0.70, r < 0.001). In partial correlation analysis, these relationships between CMM and Europeans % (r = 0.48, p < 0.001) and CMM and UVR levels (r = −0.50, r < 0.001) remained significant when GDP PPP, I_bs_, Ageing and Urbanization were statistically kept constant (Table 2–2).

Stepwise multiple linear regression analysis, including CMM incidence rate as the dependent variable, and Europeans %, UVR, GDP PPP, I_bs_, Ageing and Urbanization as the independent variables, selected UVR as the variable having the greatest but negative influence on the CMM incidence with R^2^ = 0.299; while Europeans % positive influence was placed second increasing R^2^ to 0.336 (Table 2–3).

**Table 2.** Worldwide relationships between melanoma (CMM) incidence and Europeans %.

	Table 2–1: Nonparametric		Table 2–2: Partial Correlation		Table 2–3: Stepwise multiple linear regression		
	ρ	n	r	df	Rank	Independent Variables	Adjusted R^2^
Europeans %	0.711***	127	0.477***	121	1	UVR (Negative)	0.299
UVR (Negative)	−0.699***	135	0.498***	119	2	Europeans %	0.336
GDP PPP	0.642***	129	-	-	3	I_bs_	0.400
I_bs_	0.736***	129	-	-	4	Ageing	0.470
Ageing	0.595***	130	-	-	5	GDPPPP 2010	0.487
Urbanization	0.595***	134	-	-	Not ranked	Urbanisation explained by other variables

*Note: Correlation significance level: ***p < 0.001, **p < 0.01, *p < 0.05. Data sources: Europeans % (percentage of European descendants) from the corresponding government statistics or various publications; Melanoma of skin incidence rate from the International Agency for Research on Cancer, WHO agent in cancer research; UVR, expressed as the average daily ambient ultraviolet radiation level (in J/m^2^) & ageing (life e60) from the World Health Organization; GDP PPP & Urbanization from the World Bank; Ibs from the previous publication (See the section of Data Sources please)

**Figure 3.**
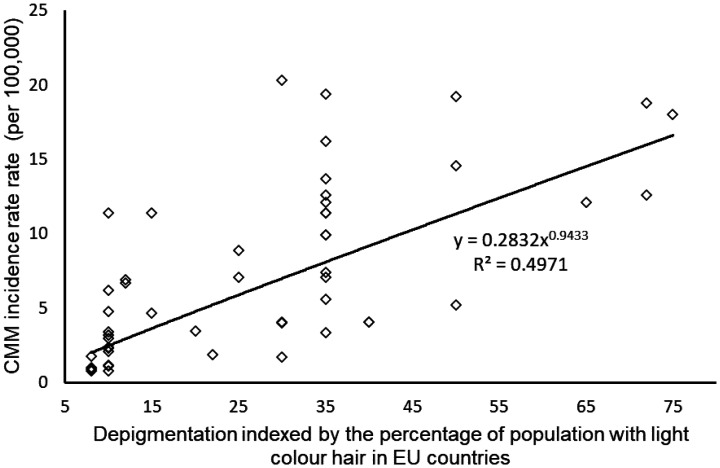
Relationship between depigmentation level and cutaneous malignant melanoma (CMM) incidence

Figure 3 indicates that country-specific depigmentation level strongly correlates with CMM incidence (Power regression line, r = 0.71, p < 0.001, n = 48).

Table 3–1 shows that CMM incidence is both in strong positive correlation with depigmentation (r = 0.70, r < 0.001, Table 3–1) and in negative correlation with UVR irradiation (r = −0.68, p < 0.001, Table 3–1). In partial correlation analysis these relationships between CMM and depigmentation (r = 0.51, r < 0.001, Table 3–2) and CMM and UVR (r = −0.43, p < 0.001, Table 3–2) remained significant when GDP PPP, I_bs_, ageing and urbanization were statistically kept constant. Depigmentation still showed significant and positive correlation with CMM (r = 0.32, p < 0.01, Table 3–3), when UVR, together with other four potential confounders (GDP PPP, I_bs_, ageing and urbanization), were included as the control variable. However, UVR showed almost nil correlation with CMM incidence rate when depigmentation, together with the other four potential confounders (GDP PPP, I_bs_, ageing and urbanization), were included as the controlled variable (Table 3–4). This indicates that, statistically, depigmentation contributes to CMM incidence rate without the contribution of UVR.

Within WHO EU, stepwise multiple regression analysis selected GDP PPP as the variable having the greatest influence on CMM incidence rate while depigmentation was placed second (Table 3–5). UVR was removed by the analysis as having no statistically significant influence on CMM incidence.

**Table 3.** Relationships between melanoma (CMM) incidence and depigmentation level within WHO Europe Region.

Table 3−1: Nonparametric (Spearman's)			Table 3−2: Partial Correlation		Table 3−3: Partial Correlation		Table 3−4: Partial Correlation		Table 3−5: Stepwise multiple linear regression (n = 50)		
	ρ	n	r	df	R	df	r	df	Rank	Independent Variables	Adjusted R^2^
Depigmentation	0.696***	48	0.512***	44	0.315**	41	-	-	1	GDP PPP	0.642
UVR (Negative)	−0.677***	50	−0.425**	42	-	-	−0.006	41	2	Depigmentation	0.720
GDP PPP	0.823***	50	-	-	-	-	-	-	3	I_bs_	0.768
I_bs_	0.769***	50	-	-	-	-	-	-	4	Ageing	Insignificant
Ageing	0.675***	50	-	-	-	-	-	-	5	Urbanization	Insignificant
Urbanization	0.631***	50	-	-	-	-	-	-	6	UVR levels	Non-predictor

*Note: Variable kept statistically constant. Data sources: Melanoma of skin incidence rate from the International Agency for Research on Cancer, WHO agent in cancer research; Pigmentation from the previous publication [74]; UVR, expressed as the average daily ambient ultraviolet radiation level (in J/m^2^) & ageing (life e60) from the World Health Organization; GDP PPP & Urbanization from the World Bank; I_bs_ from the previous publication [61]). Stepwise multiple linear regression modelling was reported. Contribution of variables is listed in order of how much they contribute to Melanoma of skin incidence. Data sources: Melanoma of skin incidence rate from the International Agency for Research on Cancer, WHO agent in cancer research; Euro-peans % (percentage of European diaspora/descendants) from the corresponding government statistics or various publications; Pigmentation from the previous publication (See the section of Data Sources please); UVR, expressed as the average daily ambient ultraviolet radiation level (in J/m^2^) & ageing (life e60) from the WHO; GDP PPP & Urbanization from the World Bank; Ibs from the previous publication (See the section of Data Sources please).

## 4. Discussion

The GLOBOCAN data on CMM incidence do not distinguish between subtypes of CMM that may have different aetiologies, and place them all under the CMM label. In our analyses the CMM label therefore includes the whole range of subtypes. According to Ward WH and Farma JM [93], the CMM subtypes occur with the following frequencies: superficial spreading (70%), nodular (5%), lentigo-maligna (4%–15%), amelanotic (4%), desmoplastic (<4%) and acral lentiginous (5%); of those, all but the last one are considered to be UV induced, thus accounting for about 95% of all CMMs [93].

Our analysis of data for 182 countries suggests that:

1) Countries with low UVR levels have high CMM incidence rates.

2) Countries with a greater percentage of European descendants have higher CMM incidence rates.

3) There is no statistical relationship between UVR level and CMM incidence per country when the percentage of European population is kept statistically constant.

4) In Europe, countries with high levels of depigmentation have higher CMM incidence rates despite lower ambient UVR levels. Country-level depigmentation negatively correlated with country-specific UVR levels providing evidence indicating that depigmentation is a long-term evolutionary adaptation to low UVR.

The magnitude of heritable depigmentation due to evolutionary adaptation to low UVR may predispose to CMM incidence worldwide, while any contribution by direct individual exposure to sunlight as a cause, however, is difficult to precisely quantify in population studies and thus effect sizes, though formally statistically significant, are low [94]–[97]. The evolutionary theory interprets how human adaptation had produced the underlying predisposition for CMM over a number of generations, likely due to arising recessive mutations/genes.

The findings of our study appear to contradict the common opinion that high UVR exposure of individual humans is the primary risk factor for CMM [6],[98]–[101]. Over generations human bodies respond to changing environmental stresses to improve their chances of adaptation, survival and reproduction. This entails better health and survival. There exist DNA repair mechanisms that remove mutagenic effects of UVR [102]. DNA methylation may also play an adaptive role [103]–[105].

Vitamin D is essential for healthy functioning of multiple body systems and organs, including bones, the lungs, cardiovascular system, immune system, and brain [107],[108]. Although UVR only constitutes approximately 10% of the total light output of the sun, it is the best natural means for producing vitamin D. Melanin pigment, produced in melanocytes, is able to dissipate more than 99.9% of UV radiation absorbed by the skin [109]. More melanin in the skin not only protects the skin cells against UV damage, but also protects against destruction of folate [60]–[62]. The natural consequence of high levels of melanin in the epidermis is inhibition of synthesis of vitamin D by the UVR [110]–[112]. People living in areas with low UVR, would be advantaged by carrying the genes/mutations which could alter their cell physiology for producing less melanin to allow better UVR penetration for balanced vitamin D genesis and adequate folate levels [61]. Vitamin D synthesis is increased by UVR, whilst folate is degraded by UVR [113]. Over generations, these mutations evolved into inheritable genetic signatures of populations with historically low UVR exposure [114],[115]. In people living for generations in areas with low-level of UVR, the amount of melanin must be balanced between allowing enough UV penetration and preventing potential solar damage to skin cells [116].

Our study suggests that CMM is not primarily caused by high UVR levels. The negative correlation between UVR and CMM across national populations indicates that historically low UVR, instead of too much UVR, may be the principal risk factor for CMM. Europeans who live in the lowest UVR levels countries have the highest CMM incidence rates based on world incidence data (Table 2, Figure 3). However, when they devloped CMM, increase of UVR could reduce their mortality. This has been revealed by an interesting study into a group of 1199 CMM patients of European ancestry whose survival is positively associated with the increase of their sun exposure [106].

Within the WHO Europe Region, CMM incidence correlates positively with depigmentation, while it correlates negatively with UVR levels. Evolutionarily, low UVR has forced Europeans to depigment, and the genetically determined depigmentation may havemade Europeans more susceptible to CMM-causing mutations. The results of our study are in agreement with the finding that some CMM subtypes can develop in skin areas with little or no UVR exposure [1],[51],[107]. A recent study has even revealed that whole-genome mutational landscapes of major CMM subtypes could occur without UVR [1]. Also, CMM may not be caused by UVR, but by xenobiotic influence [108]. CMM has been found to be familial [109] and highly heritable [110]. A number of genes predisposing to CMM have been identified [111]–[114]. A large study (N = 100,000) published in 2019, [94] found that skin colour variation within the range displayed by Norwegian women produced CMM risk ratios (RR) ranging from 1.53 to 2.32, and freckling from 2.50 to 3.30, while sun bathing produced lower RR from 0.41 to 1.71 and indoor tanning 0.85−1.18. Clearly, the risk produced by depigmentation was approximately double that resulting from UVR exposure.

A systematic review found no studies that demonstrate a causal relationship between moderate solarium use and CMM risk [55],[115]. Intermittent UVR exposure increases the risk for CMM initiation [116]–[118], but chronic exposure, for instance for outdoor workers, shows a protective role against CMM development [20],[55],[117]–[119]. Large-scale CMM prevention programmes by reduction of UVR exposure have not yet proven effective [42],[44],[120], or, unexpectedly have exacerbated CMM initiation [43],[45]–[47]. Application of sunscreen may reduce the penetration of UVR, especially UVB. This prevents sunburning, premature ageing, and non-CMM skin cancer [34],[121]. However, blocked by sunscreen, lower UVR penetration has been associated with less vitamin D_3_ genesis, leading to an increase of CMM incidence in Europeans and Americans [34],[39],[122]. Interestingly, through a randomized controlled trial, De Smedt et al. have concluded that vitamin D supplementation had a protective effect on CMM relapse, and thus, it offered patients a better clinical outcome and improved their life quality [123], especially when patients were in the advanced stages of CMM [124]. Merrill et al. have revealed that, within native populations in Europe, personal annual exposure to UVR decreased between 1960 and 2000, but CMM incidence increased significantly. This finding may indicate that lower UVR causes low vitamin D3 production leading to a CMM incidence increase [125]. The correlations between vitamin D and CMM identified in these studies may be in agreement with our hypothesis that CMM may primarily be a genetic disease of reduced pigmentation, unrelated to UVR risk. However, it should be noted that our study has been based on the population-level data and that limitation of these data is the inherent inability to assess risk behaviour at the individual personal level within those countries, for example, sunburn frequency, tanning bed usage, and individual protective behaviours. Observations of differences in CMM incidence with varying latitudes within a country are not included in the datasets we have accessed, and this aspect needs to be re-evaluated by considering the Europeans % within various parts of a country. Although, several studies within large countries have shown that latitude has low or no influence on CMM risk after correction for other confounding variables [126]–[128].

A key finding in this study that, worldwide, countries with low UVR have higher CMM incidence is opposite to conclusions from previous epidemiological studies in Australia and New Zealand. Australia and New Zealand (ANZ) have the highest CMM incidence rates internationally (34.90 and 35.80 per 100,000 population, respectively) [63], but their UVRs (3206 and 2487 J/m^2^ respectively) are not the highest in the world being comparable to Southern Europe [32] rather than equatorial Africa or central America (Figure 1).

Australians and New Zealanders (ANZ) are predominately Northern European descendants. Although there have been no clinical trials showing that high UVR causes CMM [108], there is a “consensus” that high UVR is the primary cause for CMM in ANZ. Australians and New Zealanders have learned how to seek cancer screening and to self-diagnose skin cancers. Skin cancer has been considered a “National Cancer” [129]. This strong awareness of skin cancer has enabled people to be diagnosed with more melanomas and thus, has produced increased incidence statistics. Indeed, potential over-diagnosis has been mooted [130]. Moreover, non-melanoma skin cancers (NMSC), most of which are basal cell carcinoma and squamous cell carcinoma, account for over 98% of total skin cancers. Patients with NMSC may have an increased risk for developing CMM [131]–[135] and have the highest possibility of early CMM diagnosis because their skin is clinically assessed multiple times during NMSC treatments and surveillance. The 5-year survival rate in CMM is very high (>90%) in ANZ, associated principally with earlier diagnosis of thinner CMMs, while there is a definitive chance for reoccurrence [132]. High levels of medical services and nutrition have substantially reduced natural selection. Almost all Australians and New Zealanders survive their full reproductive period, having the opportunity to pass on their CMM-related mutations/genes to the next generation. After 4–5 generations, the CMM mutations/genes accumulate and the phenotype of CMM then becomes noticeable at the population level [84],[136]. Fertility rates in ANZ are low. Low fertility rates have been associated with cancer risks in both females and males [137]–[139]. Overdiagnosis of CMM has recently again been discussed is the USA [140]. Paradoxically, long-standing advice against exposing bodies to excessive sunshine and sunbeds, has reduced “tanning” in individuals of European skin types. That is, decreased environmentally caused production of melanin in the skin, of ANZ people, so that their skin is effectively less protected from UVR penetration when accidentally exposed to sunshine. The risk of sunburn thus is more likely. The problem of high CMM incidence in ANZ is complex and clearly requires more research attention to determine the best public health advice given our data and findings.

Our hypothesis may explain why albino Africans with no melanin production (type OCA1A) do not develop CMM, while albinos with just reduced melanin production develop CMM [141]. Genetically, albinos with fully no melanin production (have no melanin), while mechanisms of melanoma causation are dependent on the genetic melanin-producing capacity [142]. This may be supported by animal (mice) experience where induction of CMM requires the presence of melanin and to be exposed to ultraviolet A (95% total UVR) [143]. Although this study has mentioned that ultraviolet B could induce CMM without requiring the presence of melanin pigment [143], it is well-established that ultraviolet B is primarily responsible for vitamin D production [119] not for CMM [35]. Albinos receive more than enough UVR for vitamin D production, under usual conditions.

It has been reported that vitamin D may protect against the development of cancers, including CMM [144]–[146] and in immune system integrity [147]. Although humans partially lost melanin production capacity (depigmentation) over generations in low UVR exposure regions for adequate vitamin D genesis, vitamin D alone may not be capable of preventing CMM occurrence. Moreover, vitamin D receptor polymorphisms perhaps associated with depigmentation have been proposed, and lower vitamin D levels have been associated with poorer CMM patient survival, which underline the complexity of vitamin D metabolism in CMM pathophysiology [148]–[150]. Also, vitamin D has been recently even associated with increased CMM rates [126], indicating that other factors may be operational and that the current story is not complete.

In our study, skin colour (reflectance) correlated with CMM incidence (r = 0.33, p = 0.057, n = 35) at a similar level, but positively, compared to the negative correlation of UVR with CMM (r = −0.52, r < 0.001, n = 171) in non-parametric analysis. However, the former correlation between armpit skin reflectance and CMM incidence lost its significance and became weak (r = 0.15, p = 0.505, df = 19) in the subsequent partial correlation. This can be explained by smaller sample size of armpit skin reflectance. Armpit skin reflectance may not be a precise measure of melanin production in the melanocytes because of a great variability of skin colour on different body sites and in different seasons [151]–[153]. Pigmentation may vary 70%–100% in the skin of the same person depending on measuring sites and seasons [154]. Therefore, pigmentation of UVR unexposed skin, such as armpit, cannot fully represent the constitutive skin pigmentation [155]–[157].

Cancers are related to somatic mutations [158]–[160]. These can occur randomly as a result of chance alterations of DNA structure that depend only on this structure's physico-chemical properties [161]–[164] while their expression may be regulated by tumour suppression [165], methylation [166], DNA repair mechanisms and immune responses [167]. Such adaptations, primarily acting to improve reproductive selective species survival advantage, may not serve to improve individual survival advantage [143]–[145],[155]. It appears that the major cause of CMM are DNA structures that evolved as adaptations to low UVR to maintain levels of vitamin D and folates. Genes for low melanin production in the normal skin may be prone to somatic mutations and methylation causing CMM.

HPV is the best-established CMM-associated retrovirus which may trigger the carriers to develop CMM as it does not only subvert immunosurveillance, but also introduces insertional mutagenesis [38]–[40]. The bivariate relationship between HPV and CMM in European populations may have confounded the correlation between UVR and CMM in Europe, however, cross-sectionally, the negative correlation between UVR and CMM incidence was identified not only in Europe, but worldwide. Longitudinally, natural selection has been acting to drive Europeans to genetically adapt for the low UVR environment. Detrimental genetic mutations inserted by HPV may partially be accumulated in the population by the modern advanced healthcare services that reduce the natural selection. Merrill et al. have suggested that the CMM increase between 1960 and 2000 could be attributed to a HPV prevalence increase during that period [125]. However, we could not locate the cross-sectional and longitudinal data on country-specific HPV prevalence or incidence rate in order to analyze the relationship between UVR and CMM incidence while ruling out the competing effect of HPV. In addition, HPV vaccination may alter HPV prevalence and subsequent analysis.

## 5. Conclusions

The main finding is that countries with low UVR levels and greater percentage of European descendants have high CMM incidence rates. No correlation between UVR level and CMM incidence is present when the percentage of European population is kept statistically constant. The results of this study therefore challenge the classical view that UVR primarily causes CMM. Our study suggests that genetic coding related to low melanin production in the skin, which evolved as a genetic adaptive trait to chronic low UVR exposure over generations, represents the primary risk factor for CMM. The depigmented European phenotype is a much higher risk than previously recognised. However, excessive sun exposure is still not recommended in view of premature ageing, sunburning and the morbidity of non-melanoma skin cancers. Considering natural selection is a dynamic process controlling genetic mutations leading to cancers, gene therapy may offer a potential approach for CMM disease control in the long run, although not immediately.
