# Supplementary material for: Cutaneous malignant melanoma incidence is strongly associated with European depigmented skin type regardless of ambient ultraviolet radiation levels: evidence from Worldwide population-based data
Source: AIMS Public Health. 2022 Mar 17;9(2):378–402. doi: 10.3934/publichealth.2022026 (PMC9114792; doi:10.3934/publichealth.2022026)
Supplement: Supplementary file 1 [file publichealth-09-02-026-s001.pdf]

Public Health

AIMS Public Health, 9(2): 378–402.
DOI: 10.3934/publichealth.2022026
Received: 28 December 2021
Revised: 25 February 2022
Accepted: 07 March 2022
Published: 17 March 2022

http://www.aimspress.com/journal/aimsph

*Research article*

# Cutaneous malignant melanoma incidence is strongly associated with European depigmented skin type regardless of ambient ultraviolet radiation levels: evidence from Worldwide population-based data

**Wenpeng You[1,*], Renata Henneberg[1], Brendon J Coventry[2] and Maciej Henneberg[1,3]**

[1] Biological Anthropology and Comparative Anatomy Unit, Adelaide Medical School, the University of Adelaide, Adelaide, South Australia, Australia
[2] Discipline of Surgery, University of Adelaide, Royal Adelaide Hospital, Adelaide, South Australia, Australia
[3] Institute of Evolutionary Medicine, University of Zurich, Zurich, Switzerland

* **Correspondence:** Email: wenpeng.you@adelaide.edu.au.

## S1. What is the Biological State Index ($I_{bs}$)?

The $I_{bs}$ calculation combines life table function $d_x$ (number of deaths at age x) with the age-specific completed relative fertility rate $S_x$ (fraction of total fertility to a woman up to age x): $I_{bs} = 1 – \Sigma d_x s_x$. More details concerning the calculation and interpretation of this index can be found in previous publications [1–6].

Due to changing mutation/ selection balance in a population, the Biological State Index ($I_{bs}$) can be considered as the index of country-specific magnitude of C43 gene accumulation [2,7].

The country-specific $I_{bs}$ was downloaded from the previous publication [4]. It has been postulated that reduced natural selection (measured by increased $I_{bs}$) may have allowed accumulation of deleterious genes of non-communicable diseases such as cancers, including C43 [2], type 1 diabetes [3] and obesity [4,8] at the population level.

## S2. Analysis of residuals

We have calculated the best fitting regression line of melanoma to each of the two variables, and then, for each country predicted its incidence using that regression. Residuals were calculated as differences between predicted melanoma incidence and its actual incidence.

These residuals were correlated with other variables in Spearman's rho analysis. They were also correlated with one another.

The best fitting equations, $y = -7.902\ln(x) + 68.305$ ($R^2 = 0.3629$) and $y = -7.902\ln(x) + 68.305$ ($R^2 = 0.3629$) were applied for the calculations of residuals of melanoma incidence standardised on Europeans % and UVR respectively.

**Table. S1** Comparisons of mean differences of melanoma (C43), UVR and Residuals of C43 standardised on UVR between WHO regions respectively.

| C43 incidence rate, Std Dev = 5.51 | | UVR level, Std Dev = 1465.29 | | Residual of C43 standardised on UVR, Std Dev = 4.50 | | WHO Region J |
|---|---|---|---|---|---|---|
| I (Region) n Mean | Mean Difference (I-J)/Std Dev* | I (Region) n Mean | Mean Difference (I-J)/Std Dev | I (Region) n Mean | Mean Difference (I-J)/Std Dev | |
| Africa (AF) | −0.16 | AF | 0.39 | AF | 0.05 | AM |
| n = 45 | 0.13 | n = 43 | 0.50$^{*}$ | n = 44 | 0.46 | EM |
| Mean = 1.13 | −1.16$^{***}$ | Mean = 5182 | 2.04$^{***}$ | Mean = −0.15 | 0.14 | EU |
| | 0.13 | | 0.13 | | 0.32 | SEA |
| | −0.39 | | 0.76$^{***}$ | | 0.11 | WP |
| Americas | 0.16 | AM | −0.39 | AM | −0.05 | AF |
| (AM) | 0.28 | n = 30 | 0.10 | n = 29 | 0.41 | EM |
| n = 32 | −1.00$^{***}$ | Mean = 4607 | 1.64$^{***}$ | Mean = −0.12 | 0.09 | EU |
| Mean = 2.01 | 0.29 | | −0.27 | | 0.27 | SEA |
| | −0.23 | | 0.37 | | 0.06 | WP |
| Eastern | −0.13 | EM | −0.50$^{*}$ | EM | −0.46 | AF |
| Mediterranean | −0.28 | n = 21 | −0.10 | n = 18 Mean = −1.18 | −0.41 | AM |
| (EM ) | −1.29$^{***}$ | Mean = 4457 | 1.54$^{***}$ | | −0.32 | EU |
| n = 22 | 0.00 | | −0.37 | | −0.13 | SEA |
| Mean = 0.44 | −0.52 | | 0.26 | | −0.34 | WP |
| Europe (EU) | 1.16$^{***}$ | EU | −2.04$^{***}$ | EU | −0.14 | AF |
| n = 50 | 1.00$^{***}$ | n = 48 | −1.64$^{***}$ | n = 50 | −0.09 | AM |
| Mean = 7.53 | 1.29$^{***}$ | Mean = 2198 | −1.54$^{***}$ | Mean = 2.00 | 0.32 | EM |
| | 1.29$^{***}$ | | −1.91$^{***}$ | | 0.18 | SEA |
| | 0.77$^{**}$ | | −1.28$^{***}$ | | −0.03 | WP |
| South-East | −0.13 | SEA | −0.13 | SEA | −0.32 | AF |
| Asia (SEA) | −0.29 | n = 11 | 0.27 | n = 10 Mean = 0.87 | −0.27 | AM |
| n = 11 | 0.00 | Mean = 4996 | 0.37 | | 0.13 | EM |
| Mean = 0.42 | −1.29$^{***}$ | | 1.91$^{***}$ | | −0.18 | EU |
| | −0.52 | | 0.63 | | −0.21 | WP |
| Western | 0.39 | WP | −0.76$^{***}$ | WP | −0.11 | AF |
| Pacific (WP) | 0.23 | n = 19 | −0.37 | n = 19 Mean = 0.83 | −0.06 | AM |
| n = 21 | 0.52 | Mean = 4070 | −0.26 | | 0.34 | EM |
| Mean = 3.18 | −0.77$^{**}$ | | 1.28$^{***}$ | | 0.03 | EU |
| | 0.52 | | −0.63 | | 0.21 | SEA |

*Note: One-way ANOVA, Bonferroni was conducted to explore the mean difference between the six WHO regions. Mean difference comparison results are reported. Significance of differences *$p < 0.05$, **$p < 0.01$, ***$p < 0.001$. Data sources: Melanoma of skin incidence rate is from the International Agency for Research on Cancer, WHO agent in cancer research; UVR, expressed as the average daily ambient ultraviolet radiation level (in $J/m^2$) is from the World Health Organization.

Average residuals of "C43 standardised on UVR" in different WHO regions do not differ between various WHO regions (Table S1). It may suggest that, without contributing effects of low UVR, the total contribution of all the other C43 risk factors may not be sufficient for the mean difference between WHO EU and other WHO Regions to reach a level of significance. This seems to indicate that without negative statistical effect of UVR on melanoma incidence (by calculating residuals), the Europeans would have the same C34 incidence as people from other regions.

## Residuals correlations

Worldwide, the residual from melanoma (C43) incidence rate standardised on Europeans % did not correlate at all with UVR in Spearman's rho analysis ($r = 0.004$, $p = 0.967$, $n = 127$). Neither did the residual from C43 incidence rate standardised on UVR correlate with Europeans % in Spearman's rho analysis ($r= -0.030$, $p = 0.735$, $n = 127$). However, the residues standardised on UVR and Europeans showed significantly strong correlation with each other ($r = 0.753$, $p < 0.001$, $n = 127$). This may suggest that, statistically, worldwide, Europeans % and UVR depend on each other for their respective correlations with melanoma incidence rate.

## References


1. Stephan CN, Henneberg M (2001) Medicine may be reducing the human capacity to survive. *Med hypothese* 57: 633–637. https://doi.org/10.1054/mehy.2001.1431
2. You W, Henneberg M (2018) Cancer incidence increasing globally: The role of relaxed natural selection. *Evol Appl* 11: 140–152. https://doi.org/10.1111/eva.12523
3. You WP, Henneberg M (2016) Type 1 diabetes prevalence increasing globally and regionally: the role of natural selection and life expectancy at birth. *BMJ Open Diab Res CA* 4: e000161. https://doi.org/10.1136/bmjdrc-2015-000161
4. Budnik A, Henneberg M (2017) Worldwide increase of obesity is related to the reduced opportunity for natural selection. *PloS One* 12:e0170098. https://doi.org/10.1371/journal.pone.0170098
5. Henneberg M, Piontek J (1975) Biological state index of human groups. *Przeglad Anthropologiczny* 41: 191–201.
6. Henneberg M (1976) Reproductive possibilities and estimations of the biological dynamics of earlier human populations. *J Hum Evol* 5: 41–48. https://doi.org/10.1016/0047-2484(76)90098-1
7. You W, Symonds I, Henneberg M (2018) Low fertility may be a significant determinant of ovarian cancer worldwide: an ecological analysis of cross-sectional data from 182 countries. *J Ovarian Res* 11: 1–9. https://doi.org/10.1186/s13048-018-0441-9

8. You W, Henneberg M (2018) Relaxed natural selection contributes to global obesity increase more in males than in females due to more environmental modifications in female body mass. *PloS One* 13: e0199594. https://doi.org/10.1371/journal.pone.0199594

AIMS Press

© 2022 the Author(s), licensee AIMS Press. This is an open access article distributed under the terms of the Creative Commons Attribution License (http://creativecommons.org/licenses/by/4.0)
